# Cardiovascular autonomic modulation and baroreflex control in the second trimester of pregnancy: A cross sectional study

**DOI:** 10.1371/journal.pone.0216063

**Published:** 2019-05-14

**Authors:** Mikaela da Silva Corrêa, Aparecida Maria Catai, Juliana Cristina Milan-Mattos, Alberto Porta, Patricia Driusso

**Affiliations:** 1 Department of Physiotherapy, Federal University of São Carlos, São Carlos, São Paulo, Brazil; 2 Department of Biomedical Sciences for Health, University of Milan, Milan, Italy; 3 Department of Cardiothoracic, Vascular Anesthesia and Intensive Care, IRCCS Policlinico San Donato, San Donato Milanese, Milan, Italy; Universidad Miguel Hernandez de Elche, SPAIN

## Abstract

**Purpose:**

The aim is to evaluate and characterize cardiovascular autonomic control and baroreflex function and their response to an orthostatic stressor in the second trimester of pregnancy via time, frequency, information and symbolic analyses.

**Methods:**

We evaluated 22 women at 18 weeks of pregnancy, labeled as pregnant group (PG) (30.8±4.4 years), and 22 non-pregnant women (29.8±5.4 years), labeled as control group (CG). Electrocardiogram, non-invasive photoplethysmographic arterial pressure (AP) and respiratory signals were recorded at rest at left lateral decubitus (REST) and during active standing (STAND) for 10 minutes. The heart period (HP) variability and systolic AP (SAP) variability were assessed in the frequency domain. High frequency (HF) and low frequency (LF) spectral indexes were computed. Nonlinear indexes such as symbolic markers (0V%, 1V%, 2LV% and 2UV% indexes), Shannon entropy (SE) and normalized complexity index (NCI) were calculated as well. Baroreflex control was assessed by cross-spectral HP-SAP analysis. We computed baroreflex sensitivity (BRS), HP-SAP squared coherence (K^2^) and phase in LF and HF bands.

**Results:**

At REST, the PG had lower mean, variance and HF power of HP series and lower K^2^(LF), BRS(LF) and BRS(HF) than the CG. During STAND, CG and PG decreased the mean, CI, NCI and 2UV% and increased 0V% of the HP series and augmented the SAP variance. LFabs of SAP series increased during STAND solely in CG. BRS(HF) was reduced during in both PG and CG, while HFabs of HP series did not diminish during STAND either in PG or CG. Complexity of the autonomic control was similar in PG and CG regardless of the experimental condition.

**Conclusion:**

We conclude that the second trimester of pregnancy was characterized by a lower parasympathetic modulation and reduced BRS at REST, preserved complexity of cardiac and vascular controls, limited sympathetic response to STAND and general conservation of the baroreflex responses to posture changes.

**Trial registration:**

Begistro Brasileiro de Ensaios clínicos, Number: RBR-9s8t88.

## Introduction

Pregnancy is a period of intense hemodynamic modifications, especially in the second trimester when cardiac output increases significantly reaching its maximum and remaining constantly elevated during the third trimester [1]. Hemodynamic changes require adjustment of mechanisms for allowing the maintenance of hemodynamic stability with the baroreflex system being the most important short-term mechanism involved in limiting the magnitude of arterial pressure (AP) oscillations [2]. The analyses of heart period (HP) variability, AP variability and baroreflex control have become powerful tools for the assessment of cardiovascular autonomic control [3–5].

The spectral analysis of HP and systolic AP (SAP) beat-to-beat fluctuations provides important information about regulation carried out by sympathetic and parasympathetic branches of the autonomic nervous system (ANS) [4]. In addition to the frequency domain indexes, symbolic and complexity analyses can provide complementary information since the cardiovascular control mechanisms interact in a nonlinear fashion and this modality of interactions cannot be fully described by traditional spectral tools [6,7]. Moreover, the analysis of the baroreflex control, via the evaluation of gain of the HP-SAP relationship linking AP changes to HP modifications, usually termed as baroreflex sensitivity (BRS), the phase (Ph) and coherence (K^2^) can provide important additional information about cardiovascular regulation. While the Ph is linked to the latency of the HP response to SAP changes, the K^2^ quantifies the degree of association between HP and SAP variabilities. Both Ph and K^2^ are highly sensitive indexes quantifying the deterioration of the ANS and baroreflex function [8].

The cardiovascular autonomic control of pregnant women has been extensively explored at rest by means of linear analyses with a certain consensus about the lower parasympathetic modulation directed to the heart and diminished BRS during gestation [9–12]. AP variability is less explored in normotensive pregnancies and appeared to be preserved at rest [13]. However, the exact mechanisms underlying the changes in autonomic cardiovascular modulation in pregnancy is not well established, especially those at the basis of the modifications of the cardiac and vascular controls. This led to controversial findings about the presumed sympathetic activation during pregnancy [14,15], as well as the preservation of the sympatho-vagal balance [13]. Also the timing at which the presumed modifications of the cardiovascular regulation occur is also controversial [1,12].

Moreover, scanty data about the complexity of cardiovascular autonomic modulation are present in literature since very few studies assessed cardiovascular control in normotensive pregnancies with nonlinear methods [5,16,17] and the studies monitoring BRS, did not report additional important parameters such as Ph and K^2^ [12,18,19]. In addition to this lack, the effect of pregnancy on hemodynamic and autonomic variables can vary according to the physical state and physiological stressor. Therefore, the response to a challenge is highly recommended [20,21]. Active standing is one of the challenges stimulating an autonomic response [21,22] that can be applied to probe the efficiency of the ANS and baroreflex controls in pregnant women.

Although some studies described attenuated BRS and HP variability after postural change in pregnancy by linear methods [9,21,23], to our knowledge, there are no reports on the autonomic modulation responses evaluated by complexity and symbolic analyses of HP and SAP variabilities and on BRS with a focus on assessing HP-SAP phase and coupling before and after a postural maneuver in pregnancy.

We hypothesize that the simultaneous use of different types of analyses and the application of a postural challenge provide additional and complementary information on the autonomic response during pregnancy, thus improving the understanding of cardiovascular regulatory mechanisms at the basis of adaptation of pregnant women and favoring the reconciliation of conflicting results present in literature. Thus, the aim of the present study was to evaluate the response of the cardiovascular autonomic modulation to active postural maneuver through different analyses in the second trimester of pregnancy.

## Methods

### Description of the population

**Fig 1:**
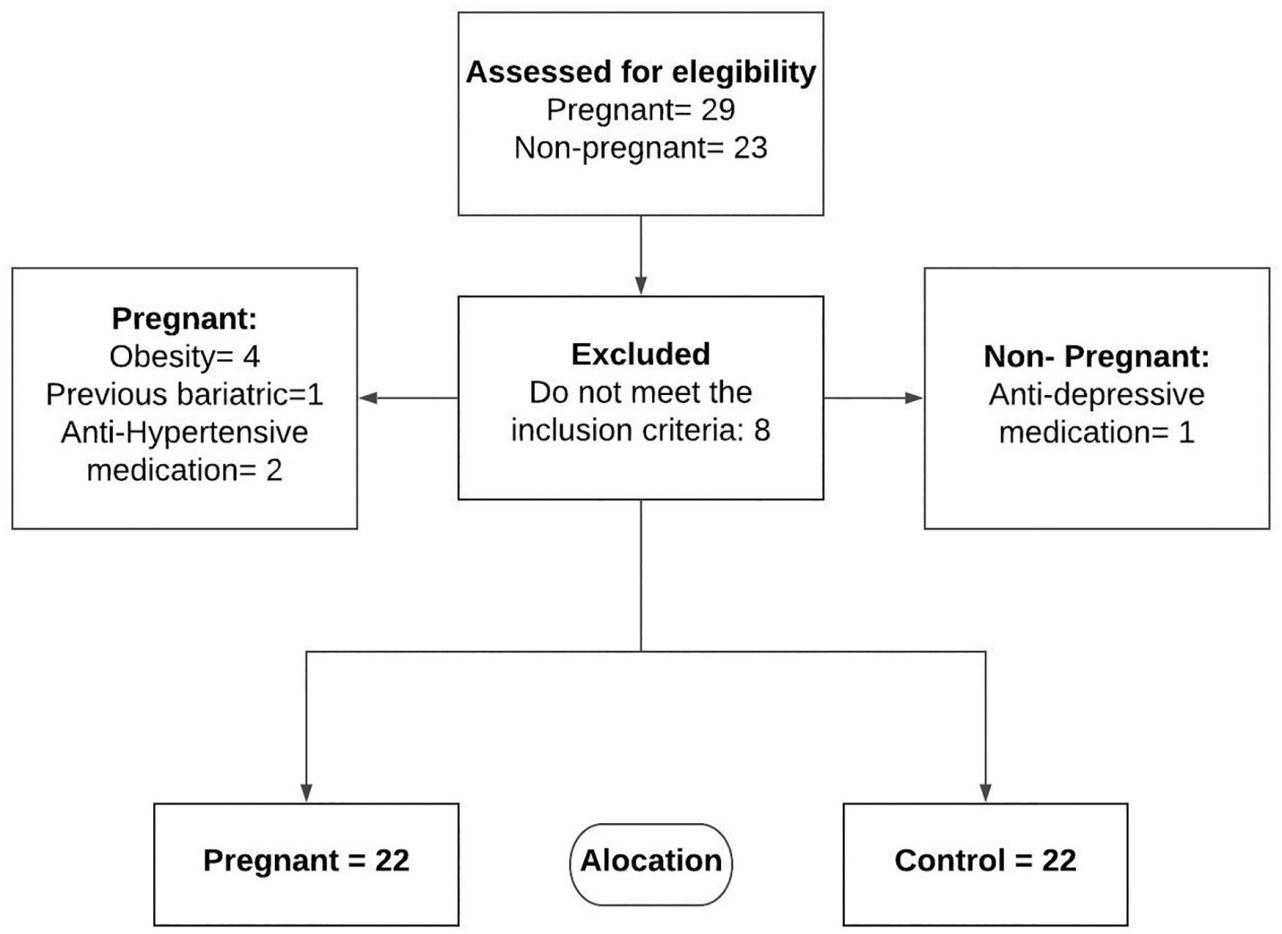
Flowchart of the enrollment. Recruitment and exclusion process.

This is a cross-sectional study that used data from a clinical trial. The clinical trial occurred between July 2015 and July 2018. Pregnant women recruited between August 2015 and July 2016, eligible to participate in the clinical trial, were selected for this study and matched by age with non-pregnant women invited to participate as a control group. Twenty nine pregnant women and 23 nulliparous women were evaluated at the Federal University of São Carlos (UFSCar) in the Physiotherapy Laboratory for Women’s Health (LAMU) and Cardiovascular Physiotherapy Laboratory (LFCV). The volunteers were recruited, informed about the proposed procedure and signed a free and informed consent term. Pregnant women were over 18 years of age, with gestational age between 18 and 22 weeks, low risk pregnancy, nonobese, and be in the first or second gestation. Nulliparous women were between 18 and 40 years of age and nonobese [24]. All volunteers were nonsmokers and did not use alcohol and illicit drugs. They were free of any cardiovascular and respiratory diseases, diabetes, hypertension, renal and liver disease, and did not use drugs that influence the AP and HP such as beta blockers, calcium channel blockers, anxiolytics, or medication for hypertension. Twenty-two women in each group were selected in this study (Fig 1). The present study was approved by the Human Research Ethics Committee of the Federal University of São Carlos, Brazil (n 1.147.092).

### Experimental protocol

The pregnant women group (PG) was evaluated between 18 and 22 weeks of gestation and compared to the nulliparous women in the control group (CG), between the 7th and 10th day of the menstrual cycle. Firstly, the volunteers underwent a standard anamnesis where they answered questions regarding their medical and obstetric history (age, delivery number and gestational age), their lifestyle habits and the use of medications. Next, the evaluation of cardiovascular autonomic modulation during resting at left lateral decubitus (REST) and after active postural change (STAND).

#### Signal acquisition setup

The volunteers were instructed to avoid alcohol and/or stimulant drinks (tea, coffee and others), strenuous exercise 24h before the evaluation, heavy meals up to 2h before the evaluation, and to have a regular sleep the night before the test. All procedures were previously explained for familiarization with the equipment and evaluators. All evaluations were performed at the same time of the day (11 am to 17 pm) in a climatically controlled room (21–23°C) and relative air humidity (40–60%). During the experiment, a minimum number of people were kept in the evaluation room. Before the evaluation, the volunteers remained at REST for 10 to 15 minutes to stabilize the cardiovascular variables and allow the equipment calibration. We recorded the electrocardiogram (ECG) via a biopotential amplifier (BioAmp FE132, ADInstruments, Australia) from the MC5 lead, noninvasive photoplethysmographic AP (Finometer-PRO, Finapres Medical System, The Netherlands) from the middle finger of the right hand and respiratory movements via a respiratory strap positioned around the thorax of the volunteers (Marazza, Monza, Italy). Signals were collected simultaneously and sampled at 1000 Hz using a commercial signal acquisition system (Power Lab 8/35, ADInstruments, Australia). Recordings were made at REST and lasted 10 minutes. Then, the volunteers were instructed to perform the postural maneuver from REST to STAND, remaining in active standing for 10 minutes. During the experiment, the volunteers were instructed to breathe spontaneously, not to talk unnecessarily and to report any changes in their physical condition (signals and/or symptoms) [25]. All participants completed the protocol.

#### Extraction of the beat-to-beat series

After detection of the QRS complex on the electrocardiogram, the peak location was performed using parabolic interpolation. The HP was calculated as the time distance between two consecutives parabolic apexes. The maximum value of AP found within an HP was considered as SAP [26]. Respiratory series was derived by sampling respiratory signal at the first QRS complex defining the onset of the HP. The occurrence of SAP and QRS complex peaks were carefully checked to avoid incorrect detections and undetected beats. Effects of ectopic beats or isolated arrhythmic events were corrected via linear interpolation. Corrections were less than 5% of the total length of the series. The measure of HP was given in milliseconds (ms) and SAP in millimeters of mercury (mmHg). Beat-to-beat series of 256 HP and SAP consecutive values were selected at REST and during STAND [27].

#### Spectral analysis of HP and SAP series

The spectral analysis of HP and SAP series was performed using an autoregressive method [28,29]. The coefficients of the autoregressive model were estimated via traditional least squares method solved recursively. The model order was optimized according to the Akaike figure of merit in the range from 8 to 14. The autoregressive spectral density was factorized into components [28,29]. A spectral component was labeled as low frequency (LF, from 0.04 to 0.15 Hz) or high frequency (HF, from 0.15 to 0.4 Hz) if its central frequency belonged to the LF or HF band. The power of the spectral components were expressed in absolute units (HFabs) or normalized units (LFnu) [4]. The normalization of the variables consisted in dividing a given spectral power expressed in absolute units by the total power minus the power below 0.04 Hz, and multiplying the ratio by 100 [28,29]. The LF power of the HP series expressed in absolute units is a marker of the sympathetic and vagal autonomic modulation directed to the heart, but when expressed in normalized units, is an index of sympathetic cardiac modulation. The HF power of HP series is a marker of vagal modulation directed to the heart [4]. The LF power of SAP series is an index of sympathetic modulation directed to the vessels. The HF power of SAP series is the result of the mechanical influence of respiration on venous return and stroke volume [30,31]. Respiratory rate was derived from respiratory series. Spectral analysis provides markers capable to described only linear dynamics. Therefore, symbolic and information domain was also applied to describe nonlinear features eventually present in HP and SAP series.

#### Symbolic analysis of HP and SAP series

Symbolic analysis was used to evaluated HP and SAP series as defined in [32]. This technique is based on the uniform quantization procedure over 6 levels applied to HP and SAP series that transforms series into sequences of symbols (from 0 to 5) from which patterns of 3 consecutive symbols were built. All possible patterns were grouped without loss into 4 families according to the number and type of variations between subsequent symbols: i) 0V pattern: all symbols are equal; ii) 1V pattern: two subsequent symbols are equal and the remaining is different; iii) 2LV pattern: the two variations between adjacent symbols have both the same sign; iv) 2UV pattern: the two variations between adjacent symbols have opposite sign. Percentages of patterns within each experimental session were evaluated and denoted with 0V%, 1V%, 2LV% and 2UV% respectively. The 0V% index was taken as a marker of the sympathetic modulation when computed over HP and SAP series and the 2UV% index was taken as an index of vagal cardiac modulation when computed over the HP series [6,7,32].

#### Information domain analysis of HP and SAP series

Shannon’s entropy (SE) and conditional entropy (CE) were performed to quantified the complexity of HP and SAP series [32]. SE quantified the complexity of the distribution of symbolic patterns. The SE is high if the distribution of pattern is flat, namely all patterns are equally distributed, while the SE is low if some patterns are more likely or others are missing [32]. The CE provides an index of dynamical complexity because it quantifies the ability of predicting the next symbol when previous ones are known. The complexity index (CI) assessing the minimal amount of complexity detected in the series according to the definition of corrected CE [32]. The normalized CI (NCI) was defined by dividing CI by SE. The NCI ranges from zero to one. NCI is 0 when series is perfectly regular, while it is 1 when irregularity is maximum [32].

#### Cross-spectral analysis of HP-SAP dynamical interactions

The cross-spectral analysis between the HP and SAP series was performed by a bivariate autoregressive model [33]. Model order was fixed to 10. The squared coherence function K^2^ was computed to quantify the degree of coupling between the HP and SAP series. It ranges from 0 to 1, where 0 indicates lack of HP and SAP correlation, and 1 indicates total association. From the transfer function we calculated the gain and Ph functions. The gain of the HP-SAP relationship was taken as an estimate of BRS expressed in ms·mmHg^-1^ and the Ph was taken as a measure of the delay between a SAP change and the corresponding response of HP expressed in radians (rad). Negative Ph indicates that HP variations lag behind SAP changes. BRS, K^2^ and Ph varied with the frequency and were sampled at the maximum coherence in the LF and HF bands [34]. The BRS, K^2^ and Ph in LF band were indicated as BRS(LF), K^2^(LF) and Ph(LF), while those in the HF band were denoted as BRS(HF), K^2^(HF) and Ph(HF) [33, 26].

#### Statistical analysis

Statistical analysis was performed using the STATISTICA software. The two-way analysis of variance was performed to evaluate the difference between experimental condition (i.e. REST and STAND) within the same group (i.e. CG or PG) and between groups within the same experimental condition. The Duncan test was performed to account for multiple comparison issue. Unpaired t-test, or Mann-Whitney rank sum test when appropriate, was utilized to evaluate the differences between parameters utilized for the general characterization of the groups. Data were expressed as mean ± standard deviation. A *p*<0.05 was taken as significant.

## Results

The characterization of the two groups is presented in Table 1. None of the reported variables differ in two groups. Five pregnant and four control women presented a body mass index (BMI) can be classified as overweight, while the remaining ones are features a normal BMI. In the PG, 8 women were in their second gestation, while 14 were primigravidae. Respiratory rates were similar regardless of groups and experimental condition.

**Table 1:** Characterization of PG and CG.

	PG n = 22	CG n = 22	*p*
Age [years]	30.78±4.37	29.83± 5.36	0.37
Weight (kg]	66.45±7.32	61±9.61	0.31
Height [m]	1.64±6.64	1.63±6.49	0.94
BMI [kg/m^2^]	24.69±2.77	23.46±3.17	0.18
Respiratory rate REST [apm]	18.68±4.39	17.82±3.87	0.37
Respiratory rate STAND [apm]	17.05±3.53	16.94±3.68	0.89
Rate of 1^th^ pregnancy	8 (36%)	-	
Rate of 2^th^ pregnancy	14 (64%)	-	

BMI: body mass index; REST: at rest at the left lateral decubitus; STAND: during active standing; apm: acts per minute. Values are expressed as mean± standard derivation or number of subjects (percentage). *p*: type I error probability.

### Linear and nonlinear analyses of HP and SAP

Over HP series at REST we found lower μ_HP_, σ^2^_HP_ and HFabs in PG (Table 2). The PG had also higher HR (Tab.2). LFnu and all the indices of complexity and symbolic analyses computed over HP series were not different between groups (Table 2). Analyses carried over SAP series at REST did not detect any between-group difference. (Table 3).

**Table 2:** HP variability markers assessed in PG and CG via time, frequency, information and symbolic analyses.

Index	Posture	PG	CG	Group effect	Posture effect	Interaction
HR [bpm]	REST	79±7	69±8*	<0.001	<0.001	<0.001
	STAND	94±10#	82±9*#			
μ_HP_ [ms]	REST	768.24±73.27	875.69±108.99*	<0.001	<0.001	<0.001
	STAND	653.28±70.15#	740.84±100.53*#			
σ^2^_HP_ [ms^2^]	REST	955.66±626.97	2021.49±1484.45*	<0.001	0.59	<0.001
	STAND	1033.12±872.94	1751.19±1569.60*			
LFnu [nu]	REST	40.79±20.69	38.53±20.55	0.36	<0.001	<0.001
	STAND	61.44±24.01#	73.34±14.85#			
HFabs [ms^2^]	REST	393.51±292.87	995.74±1020.88*	0.03	0.09	<0.001
	STAND	253.82±269.91	407.33±569.31			
SE	REST	3.69±0.37	3.64±0.04	0.33	0.03	<0.001
	STAND	3.46±0.28	3.31±0.46#			
CI	REST	1.11±0.16	1.10±0.17	0.65	<0.001	<0.001
	STAND	0.99±0.12#	0.95±0.19#			
NCI	REST	0.75±0.09	0.78±0.07	0.48	<0.001	<0.001
	STAND	0.66±0.08#	0.65±0.10#			
0V%	REST	15.38±11.17	13.20±10.29	0.89	<0.001	<0.001
	STAND	27.52±10.68#	31.40±14.13#			
1V%	REST	47.11±5.66	46.21±5.85	0.15	0.57	<0.001
	STAND	48.46±3.78	47.37±6.28			
2LV%	REST	16.75±8.74	15.34±7.99	0.58	0.09	<0.001
	STAND	11.81±6.15	10.68±7.41			
2UV%	REST	20.76±9.24	25.26±11.24	0.22	<0.001	<0.001
	STAND	12.20±5.61#	10.53±6.29#			

PG: pregnant group; CG: control group; μ_HP_: HP mean; σ^2^_HP:_ HP variance; LFnu: low frequency power expressed in normalized units; HFabs: high frequency power expressed in absolute units; CI: complexity index; NCI: normalized complexity index; 0V%: percentage of patterns with no variation; 1V%: percentage of patterns with 1 variation; 2LV%: percentage of patterns with 2 like variations; 2UV%: percentage of patterns with 2 unlike variations. Values are expressed as mean± standard deviation. SE: Shannon entropy; CI: complexity index; NCI: normalized complexity index. Values are expressed as mean± standard derivation.

The symbol * indicates a difference compared to PG group within the same experimental condition with *p*<0.05.

The symbol # indicates a difference compared to REST within in the same group with *p*<0.05.

**Table 3:** SAP variability markers assessed in PG and CG via time, frequency, information and symbolic analyses.

Index	Posture	PG	CG	Group effect	Posture effect	Interaction
μ_SAP_ [mmHg]	REST	92±9	107±13	0.20	0.19	<0.001
	STAND	107±12	100±21			
σ^2^_SAP_ [mmHg^2^]	REST	8.78±5.54	13.94±10.92	0.08	<0.001	<0.001
	STAND	19.11±13.07#	22.27±14.88#			
LFabs [mmHg^2^]	REST	4.40±3.98	5.34±9.32	0.48	0.02	<0.001
	STAND	8.29±8.20	9.80±9.50#			
LFnu [nu]	REST	72.02±16.61	68.70±21.19	0.46	0.19	<0.001
	STAND	66.67±13.59	64.62±15.57			
SE	REST	3.38±0.29	3.32±0.38	0.60	0.72	<0.001
	STAND	3.39±0.28	3.36±0.41			
CI	REST	0.95±0.12	0.93±0.16	0.66	0.78	<0.001
	STAND	0.95±0.12	0.94±0.15			
NCI	REST	0.65±0.08	0.63±0.09	0.79	0.76	<0.001
	STAND	0.63±0.07	0.64±0.08			
0V%	REST	25.05±12.11	29.20±14.19	0.55	0.98	<0.001
	STAND	27.59±9.38	26.75±15.38			
1V%	REST	52.14±4.77	50.33±6.48	0.01	0.66	<0.001
	STAND	52.68±9.38	48.80±5.96*			

PG: pregnant group; CG: control group; μ_SAP_: SAP mean; σ^2^_SAP_: SAP variance; LFabs: low frequency power expressed in absolute units; LFnu: low frequency power expressed in normalized units; SE: Shannon entropy; CI: complexity index; NCI: normalized complexity index; 0V%: percentage of patterns with no variation; 1V%: percentage of patterns with 1 variation. Values are expressed as mean± standard derivation.

The symbol * indicates a difference compared to PG group within the same experimental condition with *p*<0.05.

The symbol # indicates a difference compared to REST within in the same group with *p*<0.05.

Over HP series we found that both PG and CG responded to STAND with the decrease in μ_HP_, CI, NCI and 2UV% and increase of HR and 0V%, thus indicating a parasympathetic attenuation and greater sympathetic modulation during STAND. During STAND the PG showed lower μ_HP_ and σ^2^_HP_, and higher HR compared to CG. However, similarly to CG in PG the HFabs was not affected by STAND (Table 2), thus suggesting similar vagal withdrawal in two groups. The same conclusion held for the LFnu of HP series (Table 2).

We observed a higher σ^2^_SAP_ during STAND in both groups (Table 3). The 1V% computed over SAP increased during STAND was higher in PG than in CG and this difference was not observed at REST (Table 3). The LFabs of SAP series increased during STAND only in CG (Table 3), thus indicating a possible higher response to postural challenge in CG compared to PG.

### Cross-spectral HP-SAP indexes

K^2^(LF) was lower in PG than in CG at REST and during STAND (Table 4), thus suggesting a greater HP-SAP coupling in the CG. Conversely, BRS(LF) and BRS(HF) was lower in PG than CG but this finding held only during REST (Table 4), thus again indicating a greater sympathetic activation and lower vagal modulation limiting the magnitude of the baroreflex reaction to SAP changes in PG. K^2^(HF) and Ph(HF) did not change with group both at REST and during STAND (Table 4). Remarkably, assigned the group (i.e. PG or CG) STAND did not produce any significant modification on cross-spectral markers with the notable exception of Ph(LF) and BRS(HF): indeed, Ph(LF) was more negative at REST than during STAND in PG and BRS(HF) decreased during STAND both in PG and CG.

**Table 4:** HP-SAP cross-spectral indexes in PG and CG.

Index	Posture	PG	CG	Group effect	Posture effect	Interaction
K^2^(LF)	REST	0.70±0.14	0.79±0.13*	0.02	0.33	<0.001
	STAND	0.71±0.16	0.84±0.19*			
Ph(LF) [rad]	REST	-1.31±0.39	-1.06±0.40	0.06	0.04	<0.001
	STAND	-1.05±0.30#	-1.01±0.31			
BRS(LF) [ms/mmHg]	REST	8.55±4.95	14.08±6.59*	<0.001	0.08	<0.001
	STAND	7.82±4.71	10.70±5.26			
K^2^(HF)	REST	0.92±0.11	0.93±0.05	0.74	0.42	<0.001
	STAND	0.93±0.04	0.90±0.10			
Ph(HF) [rad]	REST	-0.46±0.44	-0.28±0.48	0.02	0.88	<0.001
	STAND	-0.52±0.44	-0.18±0.73			
BRS(HF) [ms/mmHg]	REST	18.26±9.47	30.66±16.02*	0.007	<0.001	<0.001
	STAND	9.00±7.08#	9.49±7.23#			

PG: pregnant group; CG: control group; K^2^(LF) and K^2^(HF): squared coherence in the low frequency and high frequency bands; Ph(LF) and Ph(HF): phase in the low frequency and high frequency bands; BRS(LF) and BRS(HF): baroreflex sensitivity in the LF and HF bands; rad: radians. Values are expressed as mean± standard derivation.

The symbol * indicates a difference compared to PG group within the same experimental condition with *p*<0.05

The symbol # indicates a difference compared to REST within in the same group with *p*<0.05.

## Discussion

The main findings of the present study can be summarized as follows: 1) pregnancy led to a lower vagal modulation of the heart, while sympathetic modulation to the vessels remained unmodified; 2) the response of the cardiac parasympathetic control to postural challenge is preserved in pregnancy; 3) the response of the sympathetic control to the vessels to postural challenge is lower in pregnancy; 4) the complexity of HP and SAP controls is maintained in pregnancy and responds to postural maneuver similarly to the CG; 5) pregnancy leads to a decrease of amplitude of baroreflex gain and coupling; 6) the response of baroreflex gain and coupling to STAND is preserved in PG; 7) during pregnancy the phase of the HP-SAP relationship in LF band was more negative at REST than during STAND;

### Influences of pregnancy and posture on frequency, symbolic and complexity domain parameters at REST

The lower values of σ^2^_HP_ and HFabs suggest a lower parasympathetic modulation directed to the heart in the PG at REST. The higher HR and lower HP mean are compatible with these changes. These results are in agreement with several studies that reported a lower parasympathetic contribution in pregnancy [11,15,35]. Expansion of blood volume may cause stretching of the sinoatrial node leading to a less effective vagal modulation. Animal models demonstrated a decrease in the HF power of HP variability in response to atrial stretching [36]. However, according to [9] it is unlikely that the increase of volume can fully explain the changes in the HP variability observed in a longitudinal study during pregnancy. As a matter of fact, the peak volume expansion associated with gestation occurs around 20 weeks, with little change during the third trimester, while the HF power continues to decline over this period. We suggest that adaptation to pregnancy still continue and this adaptation involves autonomic function even in absence of remarkable volume changes [9]. Thus, cardiovascular control modifications that are usually considered to be hallmarks of pathological conditions appear to be physiological in pregnancy [12]. Among the mechanisms that might lead to vagal withdrawal during pregnancy there is the activation of sympathetic branch of the ANS detected via recordings of muscle sympathetic nerve activity [37]. Even though the sympathetic activation can explain the increase of AP that occurs in the third trimester observed in some studies [37], the origin of the sympathetic overactivity is not fully elucidated. Some works advocated the activation of the renin-angiotensin system to explain the increase of sympathetic activity observed during pregnancy [38]. Some studies confirmed at the cardiac level the sympathetic activation observed at the level of muscle nerve sympathetic activity [37] with the observation the ratio of LF to HF power increased during pregnancy [14,15]. Our study LFnu did not show differences between groups, thus pointing to a preservation of the cardiac sympathetic control during pregnancy. Accordingly symbolic markers of sympathetic modulation, namely 0V% and 1V%, did not change as well. Remarkably, none of the complexity markers usually decreasing in presence of vagal withdrawal, such as CI, NCI, 2LV% and 2UV%, decreased compared to the CG, thus suggesting that further fast mechanisms, undetectable by spectral analysis, might have kept high the complexity of the cardiac vagal control during pregnancy. This finding suggests that future studies should investigate more deeply the nonlinear HP dynamics to clarify the preservation of vagal control (i.e. linear and nonlinear parts) with pregnancy in presence of a reduction of the sole linear components.

The complexity of HP and SAP variations was still poorly explored during normal gestation and utilize to detect differences between normotensive and hypertensive pregnant women [39]. As to HP analysis, some studies have reported the complexity of HP series in pregnant women at supine rest was reduced at the end of gestation [5,16]. However, in pregnant women sitting with the headboard at 45° and legs extended, the short-term HP complexity does not change before 26 weeks of gestation despite the decrease of the magnitude of HP fluctuations [17]. Even though using different method our study our result is in agreement with [17] when the same period of gestation is considered. This result indicates that factors related to the late fetus development may have triggered the decrease of HP variability complexity reported in [5,16]. As matter of fact, the effect of the supine position on venous return during pregnancy is known. The aortocaval compression of the abdominal aorta and inferior vena cava by the gravid uterus when a pregnant woman lies on her back decreases the venous return, and this may be responsible the observed modifications of makers of complexity of autonomic regulation. Indeed, the position of the body during the assessment of cardiovascular control can influence conclusions. Vagal modulation is smaller, and sympathetic modulation was higher in supine than at REST [40]. In this study we chose to adopt REST to avoid influences of aortocaval compression and we can say that the results of lower HP variability found at REST were not influenced by the mechanical compression as well as the preservation of complexity of HP control.

No studies evaluated complexity of SAP and its change in response to a stimulus in PG compared to CG. An altered SAP complexity was found in hypertensive pregnant women compared to PG [39]. We found that the SAP complexity did not change in the PG compared to CG, thus suggesting that the modifications of SAP complexity detected in [39] are not related to pregnancy but is related to hypertension or to the association between pregnancy and hypertension. Traditionally, an increase of SAP variability is exploited as early marker of gestational hypertension development [18,41]. This study did not support this use given that SAP variability did not change. Although some studies reports an increased SAP variability in the third trimester [9,42], others studies support our conclusions on the invariance of SAP variability during pregnancy [12,23,43].

### Influences of pregnancy and posture on frequency, symbolic and complexity domain parameters during STAND

STAND induces differences in both PG and CG. Both PG and CG responded to STAND by increasing LFnu and 0V% and by decreasing μ_HP_, NCI and 2UV% computed over the HP series and by increasing σ^2^_SAP_ computed over SAP series. In [21] it was found that the response of total HP variability to posture change was attenuated during gestation. However, also significant increase of HP variability during head-up tilt was reported in pregnant women [19]. In the present study, most changes in HP and SAP variability occurred in the same direction in both groups. For example, modifications of the HFabs with STAND were not significant in both groups, thus suggesting a preserved cardiac vagal response of PG to orthostatic stressor. Only the LFabs of SAP series increased significantly during STAND exclusively in CG. This finding leads us to conclude that the sympathetic activation induced by STAND was blunted in PG. This result is in agreement with previous studies detecting a smaller response of sympathetic modulation in PG in response to STAND compared with CG [9,15]. This reduced response to STAND was detected regardless whether early or late pregnancy was considered [23].

Attenuated autonomic responses may be the consequence of hemodynamic changes. In the study of Bene et al, 2001 [1], the authors describe that pregnancy from the second trimester modified the hemodynamic response to postural change, with attenuated responses of the cardiac output, HR, and peripheral vascular resistance and left ventricular ejection fraction when compared to non-pregnant women. This can be attributed to the expansion of blood volume and due to reductions in the distensibility and viscoelastic properties of the veins of the lower limb limiting modifications during posture changes [1]. In this study vascular autonomic responses to postural change are visible but of more limited amplitude. Moreover, it is worth noting that only linear indexes suggested an attenuated response. Indeed, complexity and symbolic indexes point to a preserved response of sympathetic control to postural stressor. This result suggests that pregnancy might evoke the presence on nonlinear components in the sympathetic control that might have offset the more attenuated response of linear ones. This result is more evident at the level of the vascular control given that at the level of cardiac control symbolic and complexity markers of HP series changes in agreement with the linear ones (e.g. LFnu) in response to STAND. The preserved response of complexity of HP and SAP variabilities to posture change in pregnancy may be one of the mechanisms that guarantees the efficient response of adjustments to postural change and this preservation might be utilized as a hallmark of healthy pregnancy. We remark that the preserved complexity of HP and SAP controls during STAND might be the consequence of avoiding the effects of the aortocaval compression in the supine position.

### Influences of pregnancy and posture on HP-SAP phase, coherence and BRS at REST

The analysis of the HP-SAP dynamical relationship revealed weaker HP-SAP coupling and lower BRS(LF) in PG. This means that efficiency and degree of involvement of the cardiac baroreflex in the AP regulation is decreased in pregnancy given that the magnitude of HP changes per unit variation of AP is reduced and it is less likely that HP varies in response to AP variations [8]. Both findings point to a greater difficulty in regulating AP and limiting its fluctuations in PG when compared to CG. The reduced coupling observed in this study confirmed the results relevant to synchronization analysis between HP and SAP series before and after deep breathing indicating a progressive desynchronization during the course of pregnancy [42]. The reduction of the baroreflex gain in LF band confirms the finding of other studies [9,12,44]. The decrease in BRS may be related to the reduction of vagal HP modulations observed in PG [12] as confirmed even in the present study. BRS was lower both in LF and HF bands. However, the decrease of BRS(HF) might be driven also by factors that are not of cardiac baroreflex origin [45]. Remarkably, the decrease of BRS cannot be attributed to modifications of the breathing rate given that respiratory frequency was similar in PG and CG in both experimental conditions (Tab.1). However, since during pregnancy, tidal volume rises as a consequence of a lower residual capacity even in presence of a preserved respiratory rate, additional breathing parameters need to be controlled to reliably exclude that respiration plays a role in the decrease of BRS in PG [45, 46].

### Influences of pregnancy and posture on HP-SAP phase, coherence and BRS during STAND

The effect of STAND on indexes of the baroreflex function is limited. Postural change usually causes a decrease in BRS in non-pregnant women [8]. In our study this finding was confirmed by BRS(HF) that was found to be lower after STAND in CG. In PG BRS(HF) decreased as well during STAND, thus suggesting that the response to stand was preserved. The decrease of BRS was observed also in LF band both in PG and CG but the reduction was not significant in both groups. Also this observation suggests a preservation of the baroreflex response to STAND in PG. This finding is in agreement with [19] reporting that the decrease in BRS after posture change was similar in pregnant women to non-pregnant women. The preservation of the baroreflex response to STAND in PG was stressed by the unmodified value of HP-SAP coupling after STAND. The sole results that differentiate PG from CG is that Ph(LF) was less negative during STAND than at REST in PG, while Ph(LF) was similar in CG. This result might be interpreted as an additional sign of sympathetic activation at REST in PG. Indeed, since HP was longer at REST than during STAND in PG, having a more negative Ph(LF) indicated a longer latency of the baroreflex at REST than during STAND and this result is compatible with a greater sympathetic tone [8,47].

## Limitations and future developments

The lack of hemodynamic measurements such as cardiac output does not allow confirmation of the influence of blood volume and venous return among others in the variables studied. Moreover, the lack of full monitoring of respiration prevents the ability to investigate further the effect of breathing pattern on cardiovascular control above and beyond the sole influences of breathing rate. Because the study is cross-sectional, it is possible to only state the influence of the second trimester, while preventing to follow the evolution of gestation on the cardiovascular autonomic modulation. Since the sample size was decided according to the possibility of finding a significant difference between HP mean of the pregnant group and control group according to the scheme of our protocol (two factors, namely group and experimental condition) and, then, inclusion was stopped, we advocate studies with larger sample sizes adopting the same signal processing procedure to check whether some tendencies observed in this study might become significant.

Finally, we stress the relevance of evaluating the adjustments of autonomic function and baroreflex control during pregnancy by describing not only modifications of dynamics of HP and SAP series at univariate level but also changes of their interactions through the baroreflex. This full characterization might be more powerful to predict complications and identify pregnant women at risk of developing complications. Moreover, the addition of nonlinear markers to more traditional linear indexes derived in time and frequency domains might open new possibilities of interpretation. Indeed, adjustments of cardiovascular system during pregnancy seem to preserve complexity of both cardiac and vascular controls. Given their constancy these nonlinear markers can be exploited in future studies to detect dysfunctions and deviations from normal behavior.

## Conclusions

In this study, we found that in the second trimester there is an attenuation of parasympathetic modulation, a reduced BRS and a lower coupling between HP and SAP series. These findings support a lower ability to maintain AP with changes in HP during pregnancy and a shift of the sympatho-vagal balance towards a sympathetic dominance. However, we also observed the conservation of complexity of the HP and SAP control and the preservation of the autonomic and baroreflex responses to the orthostatic challenge. This finding supports the strong resilience of the cardiac controls in pregnant women and its invariable functioning in response to a postural challenge. Only the response of sympathetic control directed to vessels with posture seems to be weaker. These findings can be helpful to better elucidate the responses of the autonomic control and baroreflex function in normal pregnancy and to detect impairment and pathological conditions in future studies.

## Supporting information

S1 Protocol Longitudinal mother study register. (PDF)

S1 Checklist STROBE checklist. (DOCX)

S1 Dataset Study data. (XLSX)
