# Supplementary material for: Cardiovascular autonomic modulation and baroreflex control in the second trimester of pregnancy: A cross sectional study
Source: PLoS One. 2019 May 14;14(5):e0216063. doi: 10.1371/journal.pone.0216063 (PMC6516729; doi:10.1371/journal.pone.0216063)
Supplement: S1 Protocol — (PDF) [file pone.0216063.s001.pdf]

# / TRIAL: RBR-9S8T88 EFEITOS CARDIOVASCULARES DA CONTRAÇÃO DOS MÚSCULOS DO PERÍNEO EM GESTANTES

## Observações

1. **Identificação do ensaio:** O "TÍTULO CIENTÍFICO" do estudo deve ser exatamente igual ao que consta no documento de aprovação pelo Comitê de Ética. Somente a primeira letra, os nomes das doenças, dos procedimentos e/ou drogas no título devem estar em caixa alta. Não deve haver pontuação no final da sentença. Exemplos: "A Efetividade da bandagem funcional em pacientes com osteoartrite de joelho - ensaio clínico randomizado: estudo piloto" ou "Estudo Fase IIb, randomizado e controlado por placebo para avaliar a eficácia clínica e segurança da Terapia de Indução e de Manutenção com BMS-936557 em indivíduos com Colite Ulcerativa (UC) ativa" ou "Alterações na expressão gênica do tecido gástrico e intestinal de pacientes diabéticos tipo 2 submetidos à Gastroplastia Redutora a Y-ROUX". Já o "TÍTULO PÚBLICO" deve estar de acordo com o título, mas em uma linguagem mais coloquial, visando o público em geral. Não utilizar termos técnicos de difícil compreensão. Por exemplo: ao invés de "cirurgia bariátrica", use "cirurgia de redução do estômago"; ou, ao invés de "alopecia", use "calvície". Somente a primeira letra, os nomes das doenças, dos procedimentos e/ou drogas no título devem estar em caixa alta. Não deve haver pontuação no final da sentença. Exemplos: Exemplo 01) Título científico: "A Efetividade da bandagem funcional em pacientes com osteoartrite de joelho - ensaio clínico randomizado: estudo piloto"; Título Público: "O efeito da bandagem elástica em pacientes com artrose de joelho"; Exemplo 02) "Estudo Fase IIb, randomizado e controlado por placebo para avaliar a eficácia clínica e segurança da Terapia de Indução e de Manutenção com BMS-936557 em indivíduos com Colite Ulcerativa (UC) ativa" ; Título Público: "Avaliação de eficácia e segurança da Terapia de Indução e de Manutenção com BMS-936557 em indivíduos com Colite Ulcerativa"; Exemplo 03) "Alterações na expressão gênica do tecido gástrico e intestinal de pacientes diabéticos tipo 2 submetidos à Gastroplastia Redutora a Y-ROUX" ; Título Público: "Alterações no tecido do estômago e intestino de pacientes diabéticos que fizeram cirurgia de redução do estômago".
Situação: Fechado

2. **Identificação do ensaio:** Prezado registrante, ao preencher o campo IDENTIFICADORES SECUNDÁRIOS, tenha em mente que é fundamental e obrigatório informar pelo menos um dos dois identificadores a seguir. 1) O número do protocolo ou registro do parecer de aprovação pelo Comitê de Ética em Pesquisa. Ou seja, não se trata do protocolo de registro/submissão/entrada do projeto de pesquisa no CEP, mas da identificação do documento emitido pelo CEP no qual consta a aprovação/aptidão do projeto para ser realizado do ponto de vista ético. Nesse caso, o "Órgão emissor" é o Comitê de Ética em Pesquisa da Instituição X. 2) Se o CEP do seu estudo está cadastrado no SISNEP ou na Plataforma Brasil, o número do CAAE (Certificado de Apresentação para Apreciação Ética) também deverá constar nos identificadores secundários. Nesse caso, o "Órgão emissor" é "Plataforma Brasil/Sistema Nacional de Ética em Pesquisa". OBS: Solicita-se ainda incluir no campo dos identificadores secundários o número do registro do estudo em outros registros de ensaios clínicos (p.ex. clinicaltrials.gov, ANZCTR etc.), se porventura realizados. OBS: Toda sigla deve também ser apresentada por extenso.
Situação: Fechado

3. **Identificação do ensaio:** No campo "Identificadores secundários", favor retirar: "UTN: U1111-1175-8972 Órgão emissor: International Clinical Trials Registry Platform (ICTRP)", pois existe um espaço específico para incluir o número do UTN.
Situação: Fechado

4. **Condições de saúde:** No campo "CONDIÇÕES DE SAÚDE OU PROBLEMAS", deve(m) ser indicado(s) a(s) condição(ões) de saúde ou problema(s) estudado(s). Por exemplo: diabetes mellitus, alopecia, obesidade etc. Se o estudo é conduzido em humanos saudáveis pertencentes à população-alvo, voluntários da intervenção (isto é, prevenção, ou intervenções de triagem), insira a condição de saúde particular(es) ou problema(s) sendo prevenido ou rastreado. Os descritores específicos que foram selecionados devem constar nesse campo, separados por vírgula ou ponto. Já para os DESCRITORES GERAIS DAS CONDIÇÕES DE SAÚDE e para os DESCRITORES ESPECÍFICOS DAS CONDIÇÕES DE SAÚDE, há dois tipos de vocabulários padronizados para informar os descritores para um estudo: o DeCS (Descritores em Ciências da Saúde) e o CID-10 (Classificação Internacional de Doenças). Tanto no campo "DESCRITORES GERAIS DAS CONDIÇÕES DE SAÚDE" como no campo "DESCRITORES ESPECÍFICOS DAS CONDIÇÕES DE SAÚDE", o registrante deverá escolher o mais adequado e os demais campos (não editáveis) serão preenchidos automaticamente. Favor, verificar se os descritores estão de acordo com as informações fornecidas pelo registrante a respeito do estudo, observando a coerência e hierarquia entre os descritores gerais e os específicos . Por exemplo: no caso de uma investigação sobre Amigdalite aguda, use o descritor geral da CID-10 'Capítulo X Doenças do aparelho respiratório' ou, no DeCS, use 'C08 Doenças Respiratórias'. Já para o descritor específico, use o CID-10 'J03 Amigdalite aguda' ou, no DeCS, use 'C07.550.781.750 Tonsilite'.
Situação: Fechado

5. **Intervenções:** No campo "INTERVENÇÕES", favor discorrer de forma clara e objetiva sobre as intervenções e procedimentos do estudo. Nesse campo deverão ser descritas todas as intervenções e os comparadores/controles que estão sendo estudados. No caso de estudos observacionais, deve-se apresentar as condições de saúde, quantidade e gênero dos integrantes, bem como os aspectos que serão observados e os procedimentos de observação. Para cada grupo de intervenção e controle, o registrante deverá detalhar o número de participantes, procedimentos, frequência, duração, modo de administração. No caso de drogas, quais as medicações foram utilizadas, o período, a quantidade e a forma como ela foi utilizada pelos sujeitos do estudo. A intervenção de controle é a intervenção contra a qual a intervenção de estudo é avaliada (p.ex. placebo, sem tratamento, controle ativo). Se um controle ativo é usado, assegure que o nome da intervenção ou as palavras "placebo" ou "sem tratamento" constem deste campo se aplicável. O registrante deverá usar o nome internacional não proprietário, não usando nomes comerciais (aceito somente para os casos quando o nome comercial seja igual ao não proprietário). Para drogas não registradas, o nome genérico, a denominação química ou número serial da companhia é aceitável. Se a intervenção consistir de vários tratamentos, o registrante deverá listar todos em uma única linha separados por vírgula (e.g. "baixo teor de gordura, dieta, exercício"). Por exemplo: "Grupo experimental:

20 mulheres com sobrepeso ou obesidade receberão refeições ricas em ácidos graxos saturados (26% do valor calórico total) acompanhadas de suco de laranja (500 mL). Grupo controle: 20 mulheres normopeso receberão refeições ricas em ácidos graxos saturados (26% do valor calórico total) acompanhadas de água (500 mL). As refeições teste e controle foram oferecidas de forma aleatorizada, com um período de lavado de 7 a 14 dias. As refeições foram feitas com pacientes em jejum". No caso de estudos multicêntricos internacionais, por favor informar o número de participantes ('n') que representa a amostra total dos sujeitos que serão submetidos a Intervenção em todos os centros e detalhar também o número de participantes incluídos na Intervenção no Brasil. O 'n' total incluindo os sujeitos de todos os centros deve constar no campo "Tamanho da amostra alvo", do menu "RECRUTAMENTO". OBS: Lembre-se que o ReBEC destina-se também ao público leigo, por esse motivo solicitamos que o seu texto tenha um número reduzido de termos técnicos (ou que sejam explicados) e que as todas siglas utilizadas sejam também esclarecidas.
Situação: Fechado

6. **Intervenções:** No campo "INTERVENÇÕES", favor discorrer de forma clara e objetiva sobre as intervenções e procedimentos do estudo. Nesse campo deverão ser descritas todas as intervenções e os comparadores/controles que estão sendo estudados. Para cada grupo de intervenção e controle, o registrante deverá detalhar o número de participantes, procedimentos, frequência, duração, modo de administração. Solicito revisão do campo em inglês, pois não está condizente com o campo em português.
Situação: Fechado

7. **Intervenções:** No campo "DESCRITORES DA INTERVENÇÃO", você deverá escolher descritores no DeCS (Descritores em Ciências da Saúde). Como no caso das condições de saúde, o registrante deverá escolher aqui o descritor mais adequado para representar as INTERVENÇÕES que serão realizadas no estudo registrado; os demais campos serão preenchidos automaticamente e não são editáveis. Verificar se os descritores das intervenções estão de acordo com as informações fornecidas pelo registrante a respeito do estudo. Para cada campo 'Descritores para as intervenções', o ReBEC oferece a possibilidade de busca no DeCS. Basta escolher a base, inserir o termo de busca e serão apresentadas ao registrante as opções existentes. Por exemplo: no caso de uma intervenção cirúrgica, é possível fazer uma busca no DeCS pelo termo 'Cirurgia' e ele apresentará 29 descritores possíveis. Cabe ao registrante selecionar aquele(s) que seja(m) representativo(s) para a intervenção pretendida no ensaio que está sendo registrado.
Situação: Fechado

8. **Recrutamento:** No campo "CRITÉRIOS DE INCLUSÃO", o registrante deverá, da forma mais concisa e objetiva possível, descrever os critérios de inclusão para seleção dos participantes, que não necessariamente são o oposto dos critérios de exclusão. Tanto em critérios de inclusão como de exclusão, retirar os marcadores de texto (letras, hífens, números, 'bolinhas', 'setinhas' etc.), não abrir parágrafo ("dar enter") e evidenciar apenas os critérios do protocolo que os sujeitos em potencial devem atender para serem elegíveis para a participação no estudo. Estes devem estar separados apenas por ponto (.) ou ponto e vírgula(;). Por exemplo: "Voluntários sadios; ambos os gêneros; não fumantes; idade entre 18 e 60 anos; peso dentro do limite de variação de 15% do peso considerado normal para homens e mulheres, levando-se em consideração altura e estrutura física.". Já no campo "CRITÉRIOS DE EXCLUSÃO", o registrante deverá descrever os critérios para a não seleção dos participantes, que não necessariamente são o oposto dos critérios de inclusão. Critérios de exclusão são diferentes de critérios de descontinuidade de um estudo. Os critérios de exclusão referem-se à seleção dos participantes para o estudo. Em critérios de inclusão e exclusão, retirar os marcadores de texto (letras, hífens, números, 'bolinhas', 'setinhas' etc.), não abrir parágrafo ("dar enter") e evidenciar apenas os critérios do protocolo que os sujeitos em potencial devem atender para serem elegíveis para a participação no estudo. Estes devem estar separados apenas por ponto (.) ou ponto e vírgula(;). Por exemplo: "voluntários fumantes; histórico de abuso de álcool ou drogas; diagnósticos de patologias cardíacas, renais, gastrintestinais, hepáticas, pulmonares, neurológicas, psiquiátricas, hematológicas ou metabólicas; antecedentes de hipersensibilidade a medicamentos; que estejam recebendo medicamentos uma semana antes do estudo ou durante a sua realização; que não estejam disponíveis para participar de todas as etapas do estudo".
Situação: Fechado

9. **Recrutamento:** No campo "Tamanho da Amostra Alvo", o registrante deverá indicar o número de participantes que este estudo pretende inscrever (ou inscreveu, no caso de recrutamento completo e/ou análise de dados completa).
Situação: Fechado

10. **Recrutamento:** Nos campos "Critérios de inclusão e exclusão", atenção a pontuação correta: Ponto e Vírgula (;) ou Ponto (.), favor não utilizar Vírgula (,).
Situação: Fechado

11. **Tipo do estudo:** Caro registrante, a revisão da seção TIPO DO ESTUDO depende diretamente das informações contidas no campo INTERVENÇÕES. Sua revisão será realizada, portanto, após os ajustes solicitados para o campo. De toda forma, de modo a otimizar o processo, pedimos especial atenção no caso de você, após realizar as alterações no campo INTERVENÇÕES, já fizer ajustes nos campos da seção TIPO DE ESTUDOS . Lembre-se de que estão disponíveis informações úteis na seção FAQ e na ajuda de campo, representada pelo sinal "?", em azul, localizado no canto esquerdo da tela de preenchimento.
Situação: Fechado

12. **Tipo do estudo:** No campo aberto "DESENHO DO ESTUDO", explicitar como o estudo foi ou será conduzido. Para essa descrição, utilize APENAS os descritores dos campos fechados selecionados pelo registrante nesta seção. A descrição deverá estar exatamente segundo as características do estudo. Perceba que o desenho de um estudo observacional É DIFERENTE do desenho de um estudo intervencional. Por exemplo: "Ensaio clínico de segurança e eficácia, randomizado, paralelo, aberto, com dois braços, de fase 2/3" ou "Estudo analítico observacional do tipo coorte/caso-controle/transversal".
Situação: Fechado

13. **Tipo do estudo:** Somente os estudos do tipo "Intervencional" com drogas deverão ter o campo "FASE DO ESTUDO" preenchido. O registrante deverá selecionar, entre as opções disponíveis (n/a, 1, 1-2, 2, 2-3, 3, 4 e 0), aquela que mais se encaixa com a fase em que se encontra seu estudo. Esta informação, assim como as demais, deverá estar de acordo com as informações fornecidas anteriormente. Para estudos observacionais a seleção será sempre n/a. No caso de estudos com drogas/medicamentos deve-se indicar a fase do estudo. A Fase 0 ou Fase pré-clínica é caracterizada pelos estudos in vitro e testes em animais. Na Fase I há o teste de um medicamento experimental pela primeira vez em um pequeno número de seres humanos saudáveis para verificar segurança, dosagem segura e efeitos colaterais. Na Fase II, o medicamento é administrado em um número maior de pessoas que têm uma doença ou condição particular, para verificar eficácia e sua segurança. A Fase III envolve centenas ou milhares de pessoas com a condição ou doença e objetiva avaliar melhor segurança e eficácia, monitorar efeitos colaterais, e compará-la a tratamentos já utilizados. Finalmente, a Fase IV consiste no monitoramento após a liberação da ANVISA e comercialização do medicamento.
Situação: Fechado

14. **Desfechos:** Tanto no campo DESFECHOS PRIMÁRIOS como no campo DESFECHOS SECUNDÁRIOS, favor rever a redação de modo a evidenciar o formato: 1) apresentação do desfecho esperado; 2) apresentação do método usado para verificar o desfecho esperado; e 3) apresentação dos parâmetros (números,

percentuais, índices etc.) utilizados para verificar se o desfecho realmente ocorreu. Por exemplo: "Aumento do número de recidivas no período de 01 ano (1) verificado por meio do método X (2) a partir da constatação de uma variação de pelo menos 5% nas medições pré e pós intervenção (3)". Use apenas um campo para cada um dos desfechos (por exemplo: apenas um campo para todos os desfechos secundários). OBS: os marcadores (1), (2) e (3) são apenas de caráter didático nesta orientação e não devem compor/aparecer o/no texto.
Situação: Fechado

15. **Desfechos:** O registrante deve inserir nesse campo o(s) desfecho(s) primário(s) esperado(s) no estudo e descrever as medidas e parâmetros utilizados na avaliação dos desfechos. Para o caso de existirem vários desfechos primários, o registrante deve eleger um principal, ou de mais importância e/ou peso. Desfechos primários são eventos, variáveis ou experiências que são medidas porque se supõe que sejam influenciadas pela intervenção em estudo. O desfecho primário é o desfecho esperado e que foi utilizado pelo pesquisador para calcular o tamanho da amostra e/ou determinar os efeitos das intervenções. São, enfim, os achados, outcomes, endpoints, os “resultados” observados ao final do estudo. Por exemplo: “Diferença média de hemoglobina sanguínea de pelo menos 6 g/L” ou “Progressão da calcificação coronariana, determinada através da tomografia coronariana no início do estudo e no final do estudo”. Caso o estudo já esteja concluído, use dois campos diferentes (quadros de texto). No primeiro quadro deverá constar os desfechos primários esperados (o que era previsto no projeto, relacionado aos objetivos e foi utilizado para o cálculo do tamanho da amostra). No segundo quadro, o registrante deverá descrever os desfechos efetivamente observados ao término do estudo.
Situação: Fechado

16. **Desfechos:** O registrante deve inserir nesse campo o(s) desfecho(s) secundário(s), caso este(s) seja(m) esperado(s) no estudo, e descrever as medidas e parâmetros utilizados na avaliação do(s) desfecho(s). Desfechos secundários são geralmente são dados que surgem durante o estudo e/ou são previstos antes do início do mesmo, e estes são usados para avaliar efeitos adicionais da intervenção. Caso, não seja esperado nenhum desfecho secundário, preencha o campo informando: "Não são esperados desfechos secundários". Por exemplo: Um desfecho secundário 1) pode envolver o mesmo evento, variável ou experiência do desfecho primário, mas medido em um período de tempo distinto do desfecho primário. Desfecho primário: mortalidade por todas as causas em 5 anos; desfecho secundário: mortalidade por todas as causas em 1 ano e 3 anos; OU 2) pode estar relacionado a um evento, variável ou experiência distinta. Desfecho primário: mortalidade por todas as causas após 5 anos; desfecho secundário: taxa de hospitalização em 5 anos. Caso o estudo já esteja concluído, use dois campos diferentes (quadros de texto). No primeiro quadro deverá constar os desfechos secundários esperados (o que era previsto no projeto, relacionado aos objetivos e foi utilizado para o cálculo do tamanho da amostra). No segundo quadro, o registrante deverá descrever os desfechos efetivamente observados ao término do estudo.
Situação: Fechado

17. **Contatos:** O registrante deverá preencher os 03 campos com informações para contato de acordo com cada tipo de caso (questões públicas, questões científicas e informações sobre o(s) centro(s) de pesquisa). Pode-se usar um mesmo contato para os 03 casos, bastando para isso preencher o formulário uma única vez e, após “salvar novo contato”, selecioná-lo nos 3 campos correspondentes. O código postal deverá ser preenchido de acordo com o país do endereço, no caso do brasil usar: xx.xxx-xxx. O telefone deverá usar o formato do país, mas sempre precedido do código do país (Brasil = +55), código da cidade (Rio = (21)) e número do telefone em questão (número de caracteres numéricos de acordo com cada país): +55 (xx) xxxx xxxx. Atenção ao fazer o registro de um contato, pois não é possível a edição de contatos. Ou seja, caso o contato seja registrado com erro, é necessário excluí-lo e realizar novo registro dos dados para o contato. (OBS: No caso de correções, faz-se necessário desvincular um contato antes de excluí-lo). A fim de facilitar a comunicação entre pesquisadores e interessados em maiores informações sobre seu estudo, solicitamos inserir o nome e os contatos do pesquisador principal (ou responsável) no campo "Contatos para questões científicas”, que consta no Parecer de Aprovação do Comitê de Ética em Pesquisa. Entendemos que, porventura, o pesquisador possa designar outra pessoa para contato científico, entretanto, julgamos pertinente também a inclusão de seus contatos no campo supracitado. No caso dos estudos multicêntricos nacionais, solicitamos inserir os contatos dos demais coordenadores do estudo no campo “Contatos para informação sobre os centros de pesquisa”. OBS: necessariamente os nomes informados em CONTATOS deverão se referir a pessoa física, ainda que ocupante de cargo/função em instituição/empresa (os dados de endereço, telefone etc. poderão ser os institucionais). OBS: como em todos os campos, as siglas deverão também ser informadas por extenso.
Situação: Fechado

18. **Contatos:** No campo "Contatos", favor preencher o campo "Endereço" completo.
Situação: Fechado

19. **Contatos:** Favor NÃO deixar o campo "Endereço" em BRANCO.
Situação: Fechado

## Respostas cardiovasculares à contração da musculatura do assoalho pélvico em gestantes

### Tipo do estudo:

Intervenções

### Título científico:

**PT-BR**
Respostas cardiovasculares à contração da musculatura do assoalho pélvico em gestantes

**EN**
Cardiovascular responses to pelvic floor muscle contractions in pregnant women

### Identificação do ensaio

**Número do UTN:** U1111-1175-8972

**Título público:**

| PT-BR | EN |
|---|---|
| Efeitos cardiovasculares da contração dos músculos do períneo em gestantes | Cardiovascular effects of the perineal muscles contractions in pregnant women |

**Acrônimo científico:**

**Acrônimo público:**

**Identificadores secundários:**

**Número do parecer: 1.147.092**
Órgão emissor: Comitê de ética em Pesquisa da Universidade Federal de São Carlos

**CAAE: 41051015.0.0000.5504**
Órgão emissor: Plataforma Brasil/Sistema Nacional de Ética em Pesquisa

## Patrocinadores

**Patrocinador primário:** Universidade Federal de São Carlos

**Patrocinadores secundários:**

**Instituição:** Universidade Federal de São Carlos

**Fontes de apoio financeiro ou material:**

**Instituição:** Universidade Federal de São Carlos

## Condições de saúde

**Condições de saúde ou problemas:**

| PT-BR | EN |
|---|---|
| Gestação, diafragma da pelve, barroreflexo, frequência cardíaca | Pregnancy, pelvic floor, barroreflex, heart rate |

**Descritores gerais para as condições de saúde:**

| PT-BR | EN |
|---|---|
| **O00-O99:** XV - Gravidez, parto e puerpério | **O00-O99:** XV - Pregnancy, childbirth and the puerperium |

**Descritores específicos para as condições de saúde:**

| PT-BR | ES | EN |
|---|---|---|
| **A01.923.600.600:** Diafragma da Pelve | **A01.923.600.600:** Diafragma Pélvico | **A01.923.600.600:** Pelvic Floor |
| **G09.330.190.400.090:** Barorreflexo | **G09.330.190.400.090:** Barorreflejo | **G09.330.190.400.090:** Baroreflex |
| **E01.370.600.875.500:** Frequência Cardíaca | **E01.370.600.875.500:** Frecuencia Cardíaca | **E01.370.600.875.500:** Heart Rate |
| **G08.686.785.760.769:** Gravidez | **G08.686.785.760.769:** Embarazo | **G08.686.785.760.769:** Pregnancy |

## Intervenções

**Categorias das intervenções**

**Behavioural**

**Intervenções:**

PT-BR

As gestantes recrutadas para esse estudo deverão ter idade entre 18 e 40 anos, índice de massa corpórea (IMC) normal para a idade gestacional, ser primigestas ou secundigestas, e apresentarer gestação de baixo risco. Também serão recrutadas mulheres não gestantes da mesma faixa etária (18- 30 anos), sedentárias, e com IMC normal para o grupo controle. Todas as mulheres não poderão apresentar presença de prolapso de órgãos pélvicos, histórico de cirurgia pélvica, uso de medicamentos que influenciem a pressão arterial e a frequência cardíaca, presença de doenças cardiovasculares, respiratórias e metabólicas, diabetes, intolerância à palpação (exame) vaginal e inabilidade em realizar contração muscular. As voluntárias incluídas neste estudo serão distribuídas em três grupos (n=24 por grupo): grupo de gestantes com treinamento (GT)- gestantes que passarão por treinamento da musculatura do assoalho pélvico (MAP) durante a gestação após o recrutamento; grupo de gestantes sem treinamento (GS)- grupo de gestantes que não realizarão o treinamento da MAP; e grupo controle (GC)- grupo de mulheres não gestantes. As voluntárias gestantes serão distribuídas nos grupos com e sem treinamento por escolha da própria voluntária, por questão ética. As participantes serão recrutadas a partir da 18ª semana de gestação, onde serão distribuídas por sua escolha nos grupos (GT ou GS). Todas as gestantes farão a primeira avaliação na 18ª semana, e as mulheres do GC farão apenas uma avaliação a ser marcada no primeiro momento do recrutamento. Primeiramente, as voluntárias serão submetidas a uma anamnese padrão, onde responderão perguntas sobre sua história ginecológica e obstétrica, seus hábitos de vida, uso de medicamentos, além da avaliação funcional da musculatura do assoalho pélvico. A avaliação funcional da musculatura do assoalho pélvico (MAP) será realizada através de palpação vaginal, parineômetria e eletromiografia. Na palpação vaginal será inserido um dedo até a segunda falange no canal vaginal, graduando-se a força

EN

The women recruited for this study must be aged between 18 and 40 years, normal body mass index (BMI) for gestational age and have a first or second low-risk pregnancy. They will also be recruited non-pregnant women of the same age (18- 30 years), sedentary, and with a normal BMI for the control group. All women may not show the presence of pelvic organ prolapse, pelvic surgery history, medications that influence blood pressure and heart rate, presence of cardiovascular, respiratory and metabolic diseases, diabetes, intolerance to palpation (examination) vaginal and inability to perform muscle contraction. The volunteers in this study will be distribuited into three groups (n = 24 per group): Group of pregnant women with training (PT) - Pregnant women who will perform pelvic floor muscles training (PFMT) during pregnancy after recruitment; group of pregnant women without training (PW) - a group of pregnant women not to perform the PMFT; and control group (CG) - the group of not pregnant women. Pregnant women will be distributed in groups with and without training by your own choosing, for ethical issue. Participants will be recruited from the 18th week of pregnancy, which will be distributed by your choice in groups (PT or PW). All pregnant women will be evaluated for the first time at 18 weeks, and women GC will be evaluated only once, to be marked on the first time of recruitment. First, the volunteers will undergo a standard medical history, which will answer questions about their gynecological and obstetrical history, your lifestyle habits, medication use, as well as functional assessment of the pelvic floor muscles. The functional evaluation of the pelvic floor muscles (MAP) will be held through vaginal palpation, perineometer and electromyography. In vaginal palpation is inserted a finger until the second phalanx in the vaginal canal, graduating muscle contraction force of PFM by the modified Oxford scale (Laycock and Jerwood; 2001). The perineometer will be inserted until the half of the probe is to 3.5cm from inside the

muscular da contração da MAP pela escala modificada de Oxford (Laycock e Jerwood; 2001). O perineômetro será inserido até que a metade da sonda fique a 3,5cm do interior do canal vaginal e serão avaliadas o pico de três contrações voluntárias máximas. Na eletromiografia, a sonda será posicionada no canal vaginal com os eletrodos na posição látero-lateral e serão captados os sinais eletromiográficos de três contrações abdominais para normalização dos dados e três contrações voluntárias máximas da MAP com duração de 5 segundos. Entre cada contração da MAP, será dado um intervalo de 1 minuto em todos os procedimentos. Entre cada equipamento ou forma de avaliação será realizado um repouso de 5 minutos.
Na 19ª semana de gestação realizarão o teste cardiopulmonar e a avaliação da modulação autonômica e monitorização fetal durante as contrações do assoalho pélvicoe mudança postural. Para análise da variabilidade da frequência cardíaca e da pressão arterial, as voluntárias serão posicionadas em decúbito lateral esquerdo e, após a calibração dos equipamentos (Finometer), serão registrados os sinais eletrocardiográficos e de pressão de pulso periférico durante 10 minutos em repouso. Após isso, deverão passar ativamente para a posição em pé, onde permanecerão por mais 10 minutos. As voluntárias deverão respirar normalmente, porém não deverão falar durante o teste. Após a mudança postural será realizada a análise durante as seguintes etapas: a) repouso de oito minutos; b) dez contrações com duração de cinco segundos e intervalo de cinco segundos entre cada contração; c) repouso de oito minutos. A Cardiotocografia será realizada simultaneamente para monitorização da vitalidade e FC fetal. Então, será realizado um teste de exercício cardiopulmonar sintoma-limitado com protocolo do tipo rampa em cicloergômetro de frenagem eletromagnética (Corival, Lode BV, Groningen, The Netherlands) na posição sentada, com flexão de joelhos entre 5 e 10 graus. O teste será iniciado com um minuto de repouso, seguido por três minutos pedalando em carga livre, e iniciado com incrementos de potência em 20W/min até a limitação por sintomas. Ao final, será realizado mais 3 minutos pedalando sem carga, seguido de um minuto de repouso. Será aferido a pressão arterial com esfigmomanômetro e as voluntárias responderam a escala de Borg a cada 1 minuto do teste.
Na 36ª semana, serão repetidas todas as

vaginal canal and will be evaluated the peak of three maximal voluntary contractions. In electromyography, the probe will be positioned in the vaginal canal with the electrodes in the lateral-lateral position and will be picked up electromyographic signals from three abdominal contractions for data normalization and three maximal voluntary contractions of PFM lasting 5 seconds. Between each contraction of the PFM is given a 1-minute interval for all procedures. Between each device or form of evaluation will be a 5 minute break. In the 19th week of pregnancy will be perform a cardiopulmonary test and evaluation of autonomic modulation and fetal monitoring during contractions of the pelvic floor and postural change. For the analysis of heart rate variability and blood pressure, the volunteers will be positioned in the left lateral position and, after calibration of equipment (Finometer) will be recorded the ECG and peripheral pulse pressure signals for 10 minutes at rest. After that, they should actively move to the standing position, where they will remain for another 10 minutes. The volunteers will breathe normally, but should not talk during the test. After postural change the analysis will be performed during the following steps: a) eight minutes rest; b) ten contractions lasting five seconds and five seconds between each contraction; c) eight minutes rest. The Cardiotocography will be held simultaneously for monitoring the vitality and fetal heart rate. So, there will be an exercise cardiopulmonary symptom-limited test with type protocol ramp electromagnetic braking cycle ergometer (Corival, Lode BV, Groningen, The Netherlands) in the sitting position with knee flexion between 5 and 10 degrees. The test will begin with a minute of rest, followed by three minutes pedaling at free of charge, and started with increments of power at 20W / min to the limitation of symptoms. At the end, there will be an additional 3 minutes pedaling without charge, followed by a minute of rest. It will be measured blood pressure with sphygmomanometer and the volunteers answered the Borg scale every 1 minute test.
At 36 weeks, will be repeated all ratings except the cardiopulmonary test. In history will be held only interview to evaluate the physical activities during this period and update the necessary data. In the 20th week of gestation the participants in the PT group will initiate the proposed training until

avaliações, exceto o teste cardiopulmonar. Na anamnese será realizada apenas a entrevista com objetivo de avaliar as atividades físicas desenvolvidas durante esse período e atualização dos dados necessários. Na 20ª semana de gestação as participantes do grupo GT iniciarão o treinamento proposto, que será feito semanalmente até a 36ª semana.
As gestantes do grupo treinamento deverão comparecer ao laboratório uma vez por semana para a realização das sessões de treinamento do assoalho pélvico (TMAP). Além do TMAP, as sessões abrangerão exercícios globais de consciência corporal, exercícios de respiração, mobilidade e relaxamento entre as posições em que serão realizadas as contrações do AP. As contrações do AP serão realizadas na posição de decúbito lateral esquerdo, sentada, ajoelhada e em pé, seguindo o seguinte protocolo em cada posição: 8 a 12 contrações; 6 a 8 segundos de sustentação da contração + 3 a 4 contrações rápidas ao final de cada contração; 6 segundos de repouso entre cada contração.
O grupo de gestantes sem treinamento, apenas realizará as avaliações na 18ª, 19ª e 36ª semanas, sem nenhuma outra intervenção. O grupo controle de mulheres não gestantes, realizará as mesmas avaliações, apenas no momento do recrutamento.

the 36th week.
The women's training group will attend the lab once a week to carry out the training sessions of the pelvic floor (TMAP). In addition to the TMAP, the sessions will cover global exercise body awareness, breathing exercises, relaxation and mobility between positions in which the contractions of the PFM will be held. PFM contractions will be held in the left lateral position, sitting, kneeling and standing, following this protocol for each position: 8-12 contractions; 6 to 8 seconds support contraction + contractions 3 to 4 fast contractions at the end of each contraction; 6 seconds of rest between each contraction.
The group of pregnant women without training, only conduct the measurements at the 18th, 19th and 36th weeks, without any other intervention. The control group of non-pregnant women, to conduct these assessments only at the time of recruitment.

**Descritores para as intervenções:**

| PT-BR | ES | EN |
|---|---|---|
| **E02.779.483:** Terapia por Exercício | **E02.779.483:** Terapia por Ejercicio | **E02.779.483:** Exercise Therapy |
| **E01.370.370.140:** Determinação da Pressão Arterial | **E01.370.370.140:** Determinación de la Presión Sanguínea | **E01.370.370.140:** Blood Pressure Determination |
| **E01.370.600.875.500:** Frequência Cardíaca | **E01.370.600.875.500:** Frecuencia Cardíaca | **E01.370.600.875.500:** Heart Rate |
| **G09.330.380.500.430:** Frequência Cardíaca Fetal | **G09.330.380.500.430:** Frecuencia Cardíaca Fetal | **G09.330.380.500.430:** Heart Rate, Fetal |
| **G03.730:** Consumo de Oxigênio | **G03.730:** Consumo de Oxígeno | **G03.730:** Oxygen Consumption |

## Recrutamento

**Situação de recrutamento:** Recruiting

**País de recrutamento**

Brazil

**Data prevista do primeiro recrutamento:** 2015-11-01

**Data prevista do último recrutamento:** 2016-11-30

| Tamanho da amostra alvo: | Gênero para inclusão: | Idade mínima para inclusão: | Idade máxima para inclusão: |
|---|---|---|---|
| 72 | F | 18 Y | 40 Y |

**Critérios de inclusão:**

PT-BR
18ª semana de gestação; faixa etária de 18 a 40 anos; índice de massa corpórea (IMC) normal para a idade gestacional de acordo com a Tabela de Atalah (Atalah SE et al., 1997); primigestas e secundigestas.

EN
18 weeks of pregnancy;age group of 18 to 40; body mass index (BMI) for gestational age according to Atalah Table (Atalah SE et al, 1997).; first or second pregnancy.

**Critérios de exclusão:**

PT-BR
Gestação de alto risco;uso de drogas; presença de prolapso de órgãos pélvicos; histórico de cirurgia pélvica; uso de medicamentos que influenciem a PA e a FC (betabloqueadores; inibidores de canais de cálcio; anti ansiolíticos); intolerância à palpação (exame) vaginal;inabilidade em realizar contração muscular ( P < 2 de acordo com método PERFECT de Laycock & Jerwood (2001); alterações no sistema cardiovascular e respiratório; doenças cardiovasculares e diabetes diagnosticadas.

EN
High-risk pregnancy; drugs use; prolapse of pelvic organs; history of pelvic surgery; taking drugs that influence BP and HR (beta blockers; calcium channel inhibitors; anti anxiety drugs); vaginal palpation Intolerance (examination);inability to perform muscle contraction (P <2 according to PERFECT method Laycock& Jerwood (2001)); changes in the cardiovascular and respiratory systems; cardiovascular and diabetes diseases diagnosed.

## Tipo do estudo

**Desenho do estudo:**

PT-BR
Estudo clínico de intervenção preventiva, paralelo, com três braços, aberto, não randomizado, prospectivo.

EN
Clinical study of preventive intervention, parallel with three arms, open, non-randomized, prospective study.

| Programa de acesso expandido | Enfoque do estudo | Desenho da intervenção | Número de braços | Tipo de mascaramento | Tipo de alocação | Fase do estudo |
|---|---|---|---|---|---|---|
| None | Prevention | Parallel | 3 | Open | Non-randomized-controlled | N/A |

## Desfechos

**Desfechos primários:**

PT-BR
Variabilidade da Frequência cardíaca (VFC) e Variabilidade da pressão arterial (VPA):

EN
Heart Rate Variability (HRV) and Blood Pressure Variability(BPV):

Essas variáveis serão avaliadas na 19ª e 36ª semana de gestação, e apenas uma vez no grupo não gestantes para se avaliar a influência da gestação, tempo gestacional, contração e treinamento da musculatura do assoalho pélvico nas variáveis.
Espera-se que a modulação autonômica seja afetada pela gestação com menor variabilidade após mudança postural e contrações do assolho pélvico em gestante, sendo essa atenuação maior no terceiro trimestre gestacional.
Os sinais eletrocardiográficos e da pressão de pulso periférico serão registrados e armazenados, batimento a batimento, durante o protocolo de exercícios perineais e mudança postural, para posterior análises da VFC e VPA.
Os dados considerados serão, no domínio da frequência (técnica linear): bandas de muito baixa frequência (MBF – entre 0 e 0,04 Hz), baixa frequência (BF – entre 0,04 e 0,15 Hz) e alta frequência (AF – entre 0,15 e 0,40 Hz). Neste estudo, utilizaremos as bandas de BF e AF, que melhor representam a modulação cardíaca simpática e vagal, respectivamente. Esses componentes espectrais serão expressos em unidades absolutas (BF e AF) e em unidades normalizadas (BFun e AFun), além da razão BF/AF (razão entre as bandas de baixa frequência e alta frequência), indicativa do balanço simpato-vagal (Task Force, 1996). Análise simbólica (técnica não linear: 1) 0V: padrão sem variação [3 símbolos iguais, por exemplo, (2,2,2) ou (4,4,4)]; 2) 1V: padrão com uma variação [2 símbolos subsequentes iguais e outro diferente, por exemplo, (4,2,2) ou (4,4,3)]; 3) 2LV: padrão com 2 variações iguais [os 3 símbolos formam uma rampa ascendente ou descendente, por exemplo, (5,4,2) ou (1,3,4)]; e 4) 2UV: padrão com 2 variações diferentes [os 3 símbolos formam um pico ou um vale, por exemplo, (4,1,2) ou (3,5,3)]. As frequências de ocorrência destas famílias (0V%, 1V%, 2LV% e 2UV%) serão avaliadas. Entropia de Shannon (técnica não linear): para fornecer uma qualificação de complexidade da distribuição dos padrões (Porta et al., 2001).
Os resultados serão analisados por comparações entre os grupos, não existindo valores de corte para caracterizar os eventos.

These variables will be evaluated at the 19th and 36th week of gestation, and only once in the non-pregnant group to evaluate the influence of pregnancy, gestational time, contracting and training of the pelvic floor muscles in the variables.
It is expected that the autonomic modulation is affected by pregnancy with less variability after postural change and contractions of the pelvic floor in pregnant women, and this greater attenuation in the third trimester.
The ECG and peripheral pulse pressure signals will be recorded and stored, beat by beat, during the protocol of perineal exercises and postural change, for further analysis of HRV and BPV.
The data will be considered in the frequency domain (linear art): very low frequency bands (MBF - between 0 and 0.04 Hz), low frequency (LF - between 0.04 and 0.15 Hz) and high frequency ( AF - between 0.15 and 0.40 Hz). In this study, we will use the bands LF and HF, which best represent the cardiac sympathetic and vagal modulation, respectively. These spectral components are expressed in absolute units (LF and HF) and in normalized units (LFnu and HFnu), and the LF / HF ratio (ratio between the bands of low frequency and high frequency), indicative of the sympathetic-vagal balance (Task Force, 1996). Symbolic Analysis (nonlinear technique: 1) 0V: no variation pattern [3 identical symbols, for example, (2,2,2) or (4,4,4)]; 2) 1V: pattern with a variation [2 following the same symbols and a different one, e.g., (4,2,2) or (4,4,3)]; 3) 2LV: 2 standard with equal variations [3 symbols form a ramp up or down, for example, (5,4,2) or (1,3,4)]; and 4) 2UV: standard with 2 different variations [3 symbols form a peak or a valley, for example, (4,1,2) or (3,5,3)]. The frequencies of occurrence of these families (0V%, 1W% 2LV% and 2UV%) will be evaluated. Shannon Entropy (nonlinear technique): to provide a qualification complexity of distribution patterns (Porta et al., 2001).
The results will be analyzed by comparisons between the groups, with no cut-off values to characterize the events.

**Desfechos secundários:**

PT-BR

Função da musculatura do assoalho pélvico.
Será avaliada na 19ª e 36ª semana de gestação, e apenas uma vez no grupo não gestantes para se avaliar a influência da gestação e tempo gestacional, e do treinamento da musculatura do assoalho pélvico na gestação.
Espera-se que o treinamento durante a gestação melhore a função muscular do assoalho pélvico.
Será avaliado: contração muscular segundo o protocolo PERFECT proposto por Laycock & Jerwood (2001) por palvação vaginal, graduando-se a função muscular pela escala de Oxford Modificada de 0 a 5.

O perineômetro Peritron device (Cardio Design Pty Ltd, Oakleigh, Victoria, Australia)possui graduação de 0 a 300 cmH2O e é equipado com uma sonda vaginal (28x55 mm). Serão realizadas três contrações do assoalho pélvico. O valor de pico médio das três contrações será utilizado para análise dos dados (Bø et al., 1999).
A eletromiografia de superfície será realizada durante contração abdominal e contração voluntária máxima (CVM)do assoalho pélvico. Para a contração abdominal e para a CVM, a média e o maior valor RMS serão computados e considerado como a atividade elétrica voluntária média e máxima, respectivamente. A atividade eletromiográfica será normalizada pela RMS média e máxima da atividade de contração adominal e pela RMS máxima da CVM.

EN

Pelvic floor muscle function.
These variable will be evaluated at the 19th and 36th week of gestation, and only once in the non-pregnant group to evaluate the influence of pregnancy, gestational time, and training of the pelvic floor muscles in pregnancy.
It is expected that the training during pregnancy improves the function of the pelvic floor muscle.
Muscle contraction will be assessed following the PERFECT protocol proposed by Laycock & Jerwood (2001) by vaginal palvação, always by the same examiner, graduating muscle function by Oxford scale modified.

The perineometer Peritron device (Cardio Design Pty Ltd, Oakleigh, Victoria, Australia) has graduation 0-300 cmH2O and is equipped with a vaginal probe (28x55 mm). Three contractions of pelvic floor muscles will be performed. The average value of the three contractions will be used for data analysis (Bø et al., 1999).
Surface electromyography will be held during abdominal contraction and maximal voluntary contraction (MVC) of the pelvic floor. For abdominal contraction and the MVC, the average and the highest RMSvalue will be counted and considered as the average and maximum voluntary electrical activity, respectively. The electromyographic activity will be normalized by the average and maximum RMS of abdominal contraction andbymaximumRMS of MVC.

PT-BR

Variabilidade da frequência cardíaca fetal.
Durante a avalição autonêmica materna também serão registrados parâmetros de VFC fetais, através da cardiotocografia para posterior análise. Espera-se encontrar sincronia entre os parâmetros maternos e fetais.

EN

Fetal Heart Rate Variability .
Variability of the fetal heart rate.
During maternal autonomic assessment will also be recorded fetal HRV parameters by cardiotocography for further analysis. We expected to find synchronicity between maternal and fetal parameters.

## Contatos

**Contatos para questões públicas**

**Nome completo:** Mikaela da Silva Corrêa

**Endereço:** Padre Teixeira, 2670, apto 101

**Cidade:** São Carlos / Brazil

**CEP:** 13560-210

**Fone:** +5516981354025

**E-mail:** mikaela.sicorrea@gmail.com

**Filiação:** Universidade Federal de São Carlos

Contatos para questões científicas

**Nome completo:** Mikaela da Silva Corrêa

**Endereço:** Padre Teixeira, 2670, apto 101

**Cidade:** São Carlos / Brazil

**CEP:** 13560-210

**Fone:** +5516981354025

**E-mail:** mikaela.sicorrea@gmail.com

**Filiação:** Universidade Federal de São Carlos

Contatos para informação sobre os centros de pesquisa

**Nome completo:** Mikaela da Silva Corrêa

**Endereço:** Padre Teixeira, 2670, apto 101

**Cidade:** São Carlos / Brazil

**CEP:** 13560-210

**Fone:** +5516981354025

**E-mail:** mikaela.sicorrea@gmail.com

**Filiação:** Universidade Federal de São Carlos

## Anexos

[Privado]

http://www.ensaiosclinicos.gov.br/static/attachments/pb_parecer_consubstanciado_cep_1147092.pdf (Parecer do comitê de ética)

OpenTrials v1.2
